# Dietary inflammatory index is associated with metabolic dysfunction-associated fatty liver disease among United States adults

**DOI:** 10.3389/fnut.2024.1340453

**Published:** 2024-03-15

**Authors:** Jing Yan, Jun Zhou, Yuanyuan Ding, Chuantao Tu

**Affiliations:** ^1^Shanghai Health Development Research Center (Shanghai Medical Information Center), Shanghai, China; ^2^School of Public Health, Fudan University, Shanghai, China; ^3^Department of Gastroenterology, Shanghai Public Health Clinical Center, Fudan University, Shanghai, China

**Keywords:** metabolic dysfunction-associated fatty liver disease, dietary inflammatory index, dietary quality, NHANES, steatotic liver disease, disease progression

## Abstract

**Background:**

Metabolic dysfunction-associated fatty liver disease (MAFLD) is presently the most prevalent chronic liver disorder globally that is closely linked to obesity, dyslipidemia metabolic syndrome, and type 2 diabetes mellitus (T2DM). Its pathogenesis is strongly associated with inflammation, and diet is a major factor in reducing inflammation. However, current research has focused primarily on exploring the relationship between diet and NAFLD, with less research on its link to MAFLD.

**Methods:**

In this research, using dietary inflammatory index (DII) as a measure to assess dietary quality, we analyzed the relationship between diet and MAFLD. Data from the National Health and Nutrition Examination Survey (NHANES) 2017–2018, including 3,633 adults with complete DII and MAFLD, were used to develop cross-sectional analyses. Logistic regression analysis was adapted for investigating the relationship between DII and MAFLD development. Additionally, subgroup analysis and threshold effect analysis were carried out.

**Results:**

A positive link between DII and MAFLD was found in the fully adjusted model (OR = 1.05; 95%CI, 1.00–1.11, *p* < 0.05). Subgroup analysis indicated that there was no significant dependence for the connection between DII and MAFLD except for the subgroup stratified by age. Compared with other age groups, people with MAFLD had 20% higher DII scores than non-MAFLD participants in those aged 20–41 years old (OR = 1.20; 95%CI, 1.08–1.33, *p* < 0.001). Furthermore, we found a U-shaped curve with an inflection point of 3.06 illustrating the non-linear connection between DII and MAFLD.

**Conclusion:**

As a result, our research indicates that pro-inflammatory diet may increase the chance of MAFLD development, thus improved dietary patterns as a lifestyle intervention is an important strategy to decrease the incidence of MAFLD.

## 1 Introduction

Non-alcoholic fatty liver disease (NAFLD) is a potentially dangerous condition that affects approximately one in four individuals worldwide, leading to a substantial burden of ill health (1, 2). NAFLD is closely linked to genetic predisposition and epigenetic or other factors including obesity, lipodystrophy, and insulin resistance (IR) or metabolic syndrome (MetS) (1, 2). Taking into account the comprehensive knowledge of NAFLD’s pathophysiology and the increasing incidence of the disease, the international panel in 2020 recommended using the metabolic dysfunction-associated fatty liver disease (MAFLD) as the replacement term for NAFLD, and further proposed more comprehensive diagnostic criteria for MAFLD (3). Following significant disputes over the previous few years, the terms steatotic liver disease (SLD) and metabolic dysfunction-associated steatotic liver disease (MASLD) were recently proposed as a replacement for NAFLD at a multi-society Delphi meeting (4).

It has been demonstrated that the new MAFLD diagnostic criterion assists in identifying those who are at high risk for cardiovascular, hepatic, and metabolic disorders and have a better application in clinical practice compared to NAFLD (5). In addition, MAFLD may also better identify patients with fatty liver and significant fibrosis diagnosed by non-invasive diagnostics (6). Previous study has shown that the MAFLD diagnostic guidelines could detect more patients who have comorbidities and poorer prognosis when compared to just the NAFLD group, which suggested that the MAFLD diagnostic criteria should be applied to the whole population in order to identify as early as possible the high-risk groups in need of intervention in high-risk populations (7).

Since there are no pharmacologically approved agents to treat MAFLD, dietary changes are crucial to preventing MAFLD development and progression (8). Treatment of MAFLD has been demonstrated to be effective when lifestyle modifications are made; nutrition, exercise, or a combination of the two may be helpful (9). It has been established that a Mediterranean diet filled with whole grains, plant-based food, fish, and olive oil lowers the risk of MAFLD by 23%, compared to a Western diet (10). Improved lifestyle adherence to the Mediterranean diet model may be beneficial for both MAFLD prevention and reversal (11). Encouraging a Mediterranean-style diet that is high in antioxidants like polyphenols, polyunsaturated fatty acids, fiber, oleic acid, and carotenoids, as well as healthier eating practices in compliance with dietary guidelines, would also be in line with lowering inflammation and the MetS while improving overall health (12). Dietary adjustment is a cost-effective strategy to mitigate the development of metabolic problems in the early stages (13). Previous research has shown that a few dietary components with antioxidant qualities can help prevent NAFLD. The amount of vitamin E and C obtained from food has an inverse relationship with how severe NAFLD is (14). Natural polyphenols found in large quantities in tea and coffee, termed chlorogenic acids, have also demonstrated to have significant anti-inflammatory and antioxidant qualities against a variety of liver conditions, including MAFLD (15).

In comparison to other indices reflecting dietary quality like healthy eating index-2015 (HEI-2015) (16), dietary inflammatory index (DII) seeks to utilize a range of study approaches and nutritional assessment methods to reflect evidence from various populations (17). DII also combines the findings from animal studies and cell culture experiments in addition to investigations involving human subjects, which increases the index’s persuasive power (18). A higher DII score leads to a more pro-inflammatory impact in diet, whereas a lower DII score enhances its anti-inflammatory effect. The DII value ranges from about −6 to +6, with the higher the number indicating less beneficial (19). Conversely, a diet is less healthy if its HEI value, which ranges from 0 to 100, is lower (16). Research previously found that among US adults, an anti-inflammatory diet, as measured by energy adjusted-DII, was linked to a reduced likelihood of developing NAFLD and early-stage fibrosis (19). According to another study, those with a more pro-inflammatory diet were more likely to acquire gastric cancer than others with a more anti-inflammatory diet (20). There is a correlation between a reduced likelihood of cancer and a diet that is higher in anti-inflammatory foods, particularly gastric and lung cancer in men, based on a prospective cohort study that has shown statistically significant and consistent relationships (21).

Of note, many of the current studies have concentrated on exploring the link between dietary factors and NAFLD development that cannot well reflect the metabolic abnormalities in this disease. Moreover, few studies provided compelling evidence that the dietary patterns that are associated with pro-inflammatory diets increased the risk of MALFD. In the current investigation, we investigated whether pro-inflammatory diets, as assessed by the DII, are linked to an elevated risk of MALFD in the adult participants of the National Health and Nutrition Examination Surveys (NHANES), which is varied in terms of race and geography.

## 2 Subjects and methods

### 2.1 Study design and population

NHANES in the US were used to collect the data. It is a national study that tracks the health and nutritional status of adults and children throughout the United States. A complicated stratified, multistage probability cluster sampling design was developed to get an accurate representation of the whole US population. The National Center for Health Statistics (NCHS) Research Ethics Review Board approved the study’s protocols. NHANES 2017–2018 data are utilized in this investigation, which for the very first time in the survey monitored liver indicators through ultrasound and vibration-controlled transient elastography (VCTE). 3,685 adults were among the 9,254 participants who signed up for this survey cycle.

**Figure 1:**
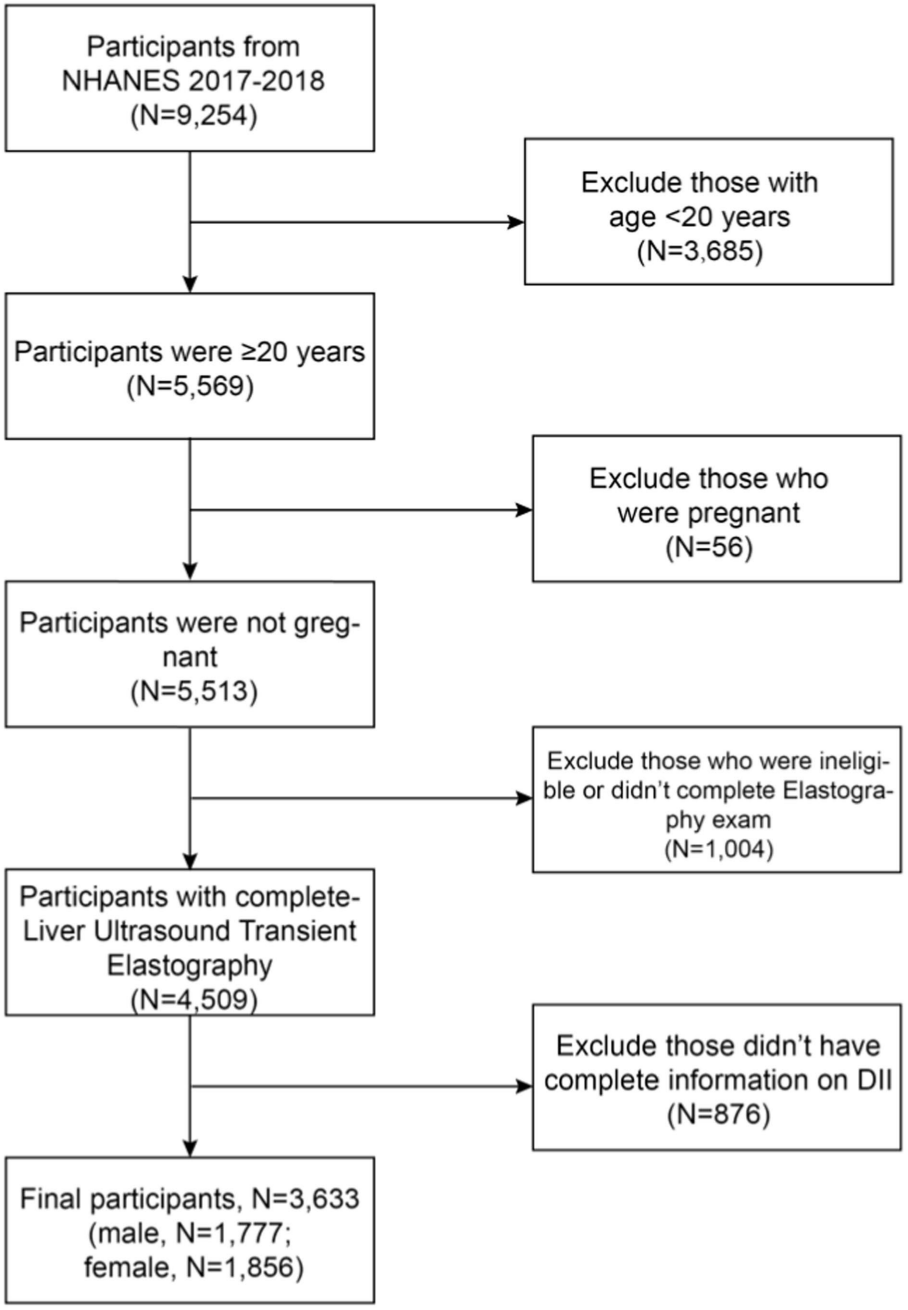
Flowchart of the selection in this study.

Following were the exclusion criteria for this cross-sectional study: (1) those who were less than 20 years old; (2) who were pregnant (a positive laboratory pregnancy test or self-reported pregnancy); (3) who were ineligible or did not complete Elastography exam; (4) missing information of DII, which were displayed in Figure 1.

### 2.2 Diagnosis of MAFLD

Participants in this study were assessed to have not passed FibroScan^®^ evaluations and were left out of the analysis if they had a fasting duration of less than 3 h, had a liver stiffness interquartile range (IQR)/median liver stiffness measurement (LSM) of over 30 percent, or had less than 10 full LSM measurements (22). A median controlled attenuation parameter (CAP) and LSM were employed, respectively, to measure liver steatosis and fibrosis. In this study, to identify hepatic steatosis, CAP 285 dB/m was selected as the cutoff threshold (23).

Histological (biopsy), imaging, or blood biomarker evidence of fat accumulation in the liver serve as the foundation for the recommended criteria for a positive MAFLD diagnosis, along with one of the following three criteria: being overweight/obese (BMI ≥ 25 kg/m^2^), suffering from type 2 diabetes mellitus (T2DM), or BMI<25 kg/m^2^ but having evidence of at least two metabolic risk abnormalities. The metabolic risk abnormalities include (1) Waist circumference 102/88 cm in men and women; (2) Blood pressure 130/85 mmHg, or particular medication treatment; (3) Plasma triglycerides 150 mg/dL or specific medication; (4) Plasma high-density lipoprotein-cholesterol (HDL-C) 40 mg/dL for men and 50 mg/dL for women; (5) Prediabetes (fast glucose 100–125 mg/dL or Hemoglobin A1c (HA1c) 5.7–6.4%); (6) Homeostasis model assessment of insulin resistance score ≥ 2.5; (7) Plasma high-sensitivity C-reactive protein (CRP) level more than 2 mg/L (3).

### 2.3 Dietary inflammatory index

The DII was created by Shivappa et al. which consists of 45 dietary components or nutrients (17). It can be utilized as a method for dietary quality evaluation to determine whether a certain diet has the potential to cause inflammation. The pro-inflammatory effect of diet develops with a greater DII score, while its anti-inflammatory effect improves with a lower DII score. The DII’s specifics are available elsewhere (17). Overall, processing raw data, calculating Z-scores, converting the centered percentiles, and multiplying the “overall inflammatory effect score” are the steps in the calculation for a specific food parameter’s DII. The sum of the DII for all food components represents the final DII score. According to the dietary interview provided by NHANES, DII was calculated using 28 out of 45 food components. The present investigation excluded elements that were a part of the initial DII computation, such as flavan-3-ol, flavones, flavonols, flavonones, anthocyanidins, isoflavones, pepper, thyme/oregano, and rosemary, since the NHANES 2017–2018 data did not have them accessible. The majority of the food items that are absent are often consumed in little amounts by this population, and therefore their absence is probably not going to have a big impact on the overall DII ratings. The ultimate result of DII was calculated by two 24-h dietary recall interview data.

### 2.4 Covariates

Covariates that may affect the relationship between DII and MAFLD were included in our study as well, including age, gender, race, education, ratio of family income to poverty (PIR), body mass index (BMI), LSM, hypersensitive C reactive protein (HSCRP), HbA1c, alanine aminotransferase (ALT), aspartate aminotransferase (AST), Albumin, minutes of sedentary activity, hypertension, diabetes, drug use.

Education level was classified into below high school, high school graduate, some college or AA degree, college graduate or above. Low level (PIR < 1.30), middle level (1.30 ≤ PIR < 3.50), and high level (PIR ≤ 3.50) were the three levels of the poverty income ratio (PIR), which was used to determine the levels of household income (24). In addition, drug use was classified into never, moderate (<50 times), and heavy (≥50 times).

### 2.5 Statistical analysis

For all statistical analyses, we applied SPSS Statistics 26 and EmpowerStats (version 2.0), with statistical significance set at *p* < 0.05. To account for missing data on BMI, HSCRP, HbA1c, ALT, AST, Albumin, PIR, and minutes of sedentary activity, we utilized multiple imputations, based on 5 replications in the SPSS.

According to the analytical standards of NCHS, sample weights were employed to compute all estimates in order to represent the US population. Three models were used to construct the logistic regression: model 1, with no factors adjusted; model 2, with age, gender, race, and education adjusted; model 3, with all covariates corrected. Subgroup analyses were also carried out. The link and inflection point between DII and MAFLD was investigated by using a threshold effects analysis model.

## 3 Results

### 3.1 Baseline characteristics of participants

According to the inclusion and exclusion criteria, 3,633 individuals were included in the research. They were classified into non-MAFLD and MAFLD groups according to the recommended standard. Age, gender, race, education, DII, BMI, LSM, HSCRP, TC, HbA1c, ALT, AST, Albumin, minutes of sedentary activity, hypertension, diabetes, and use of drugs were all statistically different among the two groups (all *p* < 0.05). With regard to DII score Quartile 1 (−5.10 ~ −0.27), a smaller proportion of participants with MAFLD (23.08%) had it compared to those with non-MAFLD (27.75%). The overall features of individuals who participated are shown in Table 1 according to MAFLD category.

**Table 1:** Characteristics of participants, weighted.

	Non-MAFLD (*N* = 2,255)	MAFLD (*N* = 1,378)	*p* Value
Age (years)	45.88 ± 17.27	51.56 ± 16.05	<0.0001
Gender (%)			<0.0001
Male	44.76	55.72	
Female	55.24	44.28	
Race (%)			<0.0001
Mexican American	6.48	12.52	
Other Hispanic	6.57	6.93	
Non-Hispanic White	63.47	61.86	
Non-Hispanic Black	12.89	8.92	
Other Race	10.58	9.77	
Education level (%)			<0.0001
Below high school	8.92	10.41	
High school graduate	25.28	29.78	
Some college or AA degree	29.12	33.43	
College graduate or above	36.68	26.38	
PIR (%)			0.4255
<1.30	19.17	20.29	
≥1.30, <3.50	35.53	36.61	
≥3.50	45.30	43.10	
DII (%)			<0.0001
Quartile 1 (−5.10 ~ −0.27)	27.75	23.08	
Quartile 2 (−0.26 ~ 1.07)	27.69	25.94	
Quartile 3 (1.08 ~ 2.27)	20.32	27.73	
Quartile 4 (2.28 ~ 4.82)	24.25	23.24	
DII	0.79 ± 1.77	1.00 ± 1.62	0.0006
LSM (Kpa)	5.01 ± 3.52	6.83 ± 6.75	<0.0001
BMI (kg/m^2^)	27.16 ± 5.74	33.84 ± 6.54	<0.0001
HSCRP (mg/L)	2.96 ± 6.84	5.11 ± 8.26	<0.0001
TC (mg/L)	187.90 ± 38.82	192.80 ± 42.26	0.0004
HbA1c (%)	5.46 ± 0.61	6.07 ± 1.25	<0.0001
ALT (U/L)	20.79 ± 14.88	28.96 ± 20.46	<0.0001
AST(U/L)	22.28 ± 14.92	23.74 ± 12.51	0.0027
Albumin (g/dl)	4.13 ± 0.31	4.08 ± 0.31	<0.0001
Minutes of sedentary activity (min)	337.25 ± 188.77	361.65 ± 202.47	0.0003
Hypertension (%)			<0.0001
No	88.97	80.06	
Yes	11.03	19.94	
Diabetes (%)			<0.0001
No	95.34	79.92	
Yes	4.66	20.08	
Drinking status (%)			0.1259
Low	68.42	71.41	
Moderate	24.31	21.38	
Heavy	7.27	7.21	
Drug use (%)			<0.0001
Never	96.05	98.95	
Moderate	2.49	0.38	
Heavy	1.45	0.66	

PIR, ratio of family income to poverty; BMI, body mass index; LSM, a median Liver Stiffness Measurement; HSCRP, hypersensitive C reactive protein; TC, cholesterol; HbA1c, Glycohemoglobin; ALT, alanine aminotransferase; AST, aspartate aminotransferase.

### 3.2 The relationship between DII and MALFD

Our findings indicated that a higher DII was associated with an increased risk of MAFLD. This connection was statistically significant both in the minimally adjusted model (OR = 1.09; 95%CI, 1.04–1.13, *p* < 0.001) and the fully adjusted model (OR = 1.05; 95%CI, 1.00–1.11, *p* < 0.05). The outcome of the fully adjusted model revealed a 5% increase in the likelihood of MAFLD for every unit higher DII value. For the sensitivity analysis, we further transformed the continuous variable DII into a categorical variable (quartiles; Table 2).

**Table 2:** Association of DII with MAFLD.

Exposure	OR (95%CI), *p* value		
	Model 1^a^	Model 2^b^	Model 3^c^
DII	1.04 (1.00, 1.08) 0.0589	1.09 (1.04, 1.13) 0.0002	1.05 (1.00, 1.11) 0.0474
Quartile of DII			
Q1(−5.10 ~ −0.27)	1.0	1.0	1.0
Q2(−0.26 ~ 1.07)	1.01 (0.84, 1.23) 0.8861	1.06 (0.87, 1.30) 0.5351	0.95 (0.76, 1.20) 0.6726
Q3(1.08 ~ 2.27)	1.30 (1.08, 1.57) 0.0066	1.45 (1.19, 1.78) 0.0002	1.27 (1.01, 1.61) 0.0436
Q4(2.28 ~ 4.82)	1.11 (0.92, 1.34) 0.2894	1.36 (1.10, 1.67) 0.0037	1.18 (0.92, 1.50) 0.1865
*p* Value for trend	0.0693	0.0002	0.0453

^a^No-adjusted model: adjusted for none. ^b^Minimally adjusted model: adjusted for age, gender, race, and education. ^c^Fully adjusted model: adjusted for age, gender, race, education, PIR, BMI, LSM, HSCRP, HbA1c, ALT, AST, Albumin, minutes of sedentary activity, hypertension, diabetes, and drug use.

### 3.3 Subgroup analysis

A statistically significant correlation between DII and MALFD was found for the subgroup that was age-stratified (*p* for interaction<0.05). Compared with other age groups, people with MAFLD had 20% higher DII scores than non-MAFLD participants in those aged 20–41 years old (OR = 1.20; 95%CI, 1.08–1.33, *p* < 0.001; Table 3).

**Table 3:** Stratified logistic regression analysis to identify variables that modify the correlation between DII and MAFLD*.

	OR (95%CI), *p* value	*p* for interaction
Gender		0.4691
Male	1.03 (0.96, 1.11) 0.3836	
Female	1.07 (1.00, 1.15) 0.0601	
Age (years)		0.0137
20–41	1.20 (1.08, 1.33) 0.0006	
42–60	1.06 (0.98, 1.16) 0.1637	
61–80	0.99 (0.92, 1.07) 0.8019	
Race		0.3498
Mexican American	1.03 (0.89, 1.19) 0.7246	
Other Hispanic	0.99 (0.85, 1.15) 0.9008	
Non-Hispanic White	0.99 (0.91, 1.08) 0.8073	
Non-Hispanic Black	1.14 (1.02, 1.27) 0.0198	
Other Race	1.01 (0.90, 1.13) 0.8561	
Education		0.5156
Below high school	1.05 (0.92, 1.18) 0.4757	
High school graduate	1.01 (0.91, 1.13) 0.8416	
Some college or AA degree	1.11 (1.02, 1.22) 0.0213	
College graduate or above	1.02 (0.93, 1.13) 0.6299	
PIR		0.1243
<1.30	0.99 (0.91, 1.08) 0.8392	
≥1.30, <3.50	1.12 (1.03, 1.21) 0.0049	
≥3.50	1.05 (0.95, 1.16) 0.3240	
Minutes of sedentary activity (min)		0.9347
0–234.53	1.05 (0.96, 1.15) 0.2629	
240–356.41	1.07 (0.97, 1.18) 0.1656	
360–1,320	1.05 (0.97, 1.13) 0.2538	
BMI		0.1668
<25	1.06 (0.92, 1.24) 0.4111	
≥25, <30	1.00 (0.92, 1.08) 0.9353	
≥30	1.11 (1.03, 1.19) 0.0061	

*The results of subgroup analysis were adjusted for all covariates (age, gender, race, education, PIR, BMI, LSM, HSCRP, HbA1c, ALT, AST, Albumin, minutes sedentary activity, hypertension, diabetes, drug use) except for effect modifier.

### 3.4 Non-linearity and saturation effect analysis between DII and MAFLD

**Figure 2:**
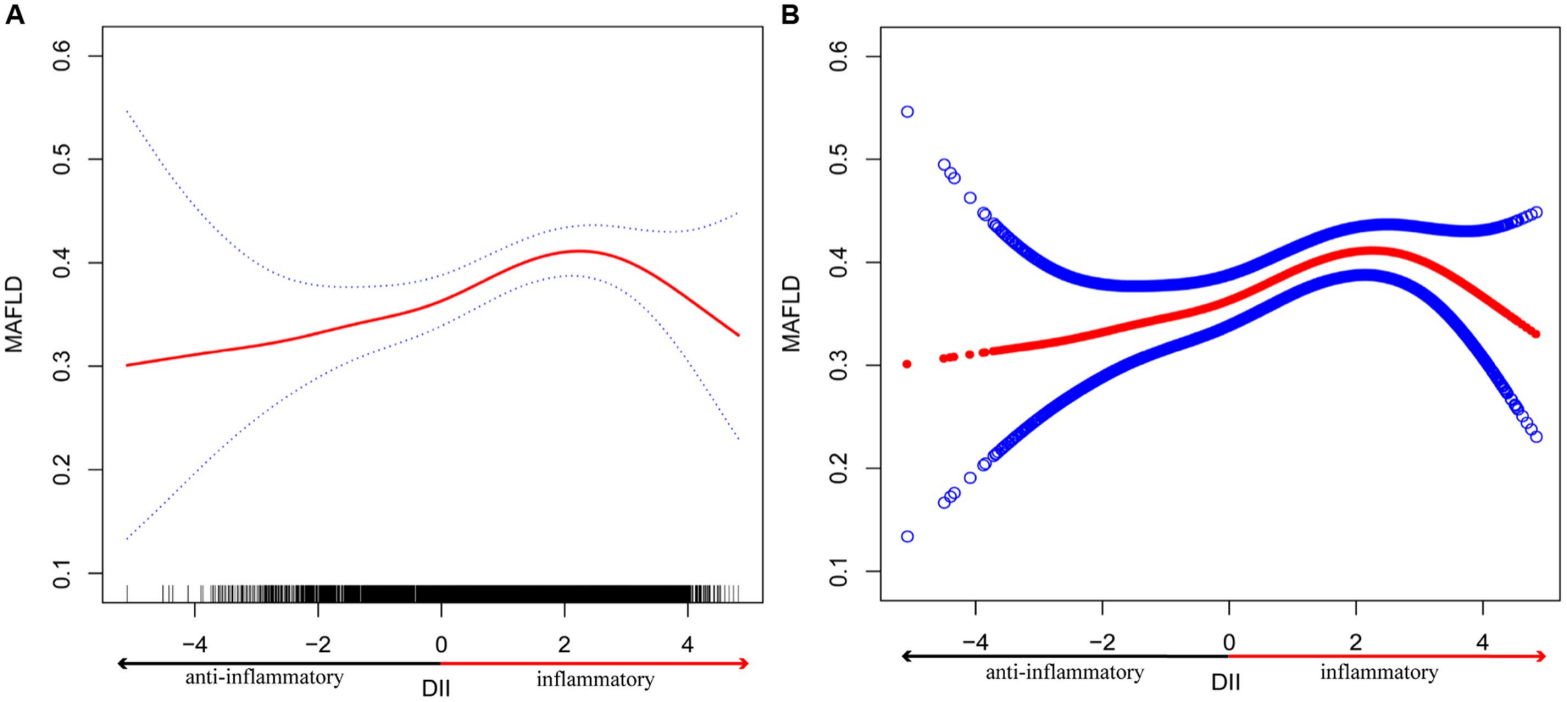
The association between DII and MAFLD. **(A)** The smooth curve fit between variables is represented by the solid red line. The 95% confidence interval from the fit is represented by blue bands. **(B)** The solid red line represents the smooth curve fit between variables. Blue bands represent the 95% confidence interval from the fit. *Age, gender, race, education, PIR, BMI, LSM, HSCRP, HbA1c, ALT, AST, Albumin, minutes of sedentary activity, hypertension, diabetes, and drug use were adjusted.

In order to better understand the nonlinear link that exists between DII and MAFLD, we fitted a smooth curve (Figure 2). We discovered an inverted U-shaped correlation between DII and MAFLD through a two-segment logistic regression model, with an inflection point of 3.06 (Table 4). When the DII was less than 3.06, a significant positive correlation between DII and MAFLD was discovered; the regression coefficient was 1.09 (95%CI, 1.03–1.16; *p* = 0.0022), indicating a 9% increase in the likelihood of MAFLD for each SD increase in DII. However, if the DII was higher than 3.06, a negative correlation between DII and MAFLD was discovered; the regression coefficient was 0.56 (95%CI, 0.36–0.86; *p* = 0.0089), demonstrating a 44% decrease in the likelihood of MAFLD for each SD increase in DII.

**Table 4:** Threshold effect analysis of DII on MAFLD using two-piecewise logistic regression model*.

MAFLD	Adjusted OR (95% CI) *p* value
*DII*	
Inflection point	3.06
DII < 3.06	1.09 (1.03, 1.16) 0.0022
DII > 3.06	0.56 (0.36, 0.86) 0.0089
Log likelihood ratio	0.003

*Age, gender, race, education, PIR, BMI, LSM, HSCRP, TC, HbA1c, ALT, AST, Albumin, minutes of sedentary activity, hypertension, diabetes, and drug use were adjusted.

## 4 Discussion

We found a positive association between DII and MAFLD in this cross-sectional investigation, including 3,633 participants. Our data also demonstrated that the correlation was not significantly influenced by gender, race, education, PIR, minutes of sedentary activity, and BMI, suggesting that an increased likelihood of MAFLD may result from higher DII. Besides, MAFLD patients had higher DII scores than the non-MAFLD participants for individuals between the ages of 20 and 41, compared to adults of other age groups. Of note, between DII and MAFLD, an inverted U-shaped relationship with a 3.06 inflection point was identified. Our present study suggested that improving dietary quality might be a reduced risk of developing MAFLD. In account of these findings, it is recommended that people eat less food high in fat, cholesterol, and carbohydrates and more foods rich in nutrients that reduce inflammation, such as dietary fiber, thiamin, riboflavin, niacin, magnesium, and zinc. It is particularly crucial to focus on maintaining a healthy diet for individuals between the ages of 20 and 41, and a dietary pattern with a low DII score should be followed.

As far as we are aware, this is the first comprehensive study to clarify the relationship between DII and MAFLD development based on NHANES information. Prior study has demonstrated a positive correlation between DII and the phenotypes of MAFLD (25), but the main emphasis of that study lays in studying the association between the DII and other dietary assessment indices; no threshold effect analyses or subgroup analyses of the DII and MAFLD were performed. A Sabzevar Persian cohort study has also reported that greater exposures to pro-inflammatory dietary may be linked to an increased risk of MAFLD in adults (26). Better food quality is related to a reduced likelihood of MAFLD, according to other research using dietary indices like HEI-2015 (27, 28). Another prospective study found that eating a healthy diet was linked to a decreased chance of developing fatty liver, especially when the fatty liver was associated with biochemical alterations (29). The study also discovered that changing one’s diet might lessen one’s genetic predisposition to MAFLD. Despite the fact that earlier study has connected higher DII scores to an elevated likelihood of developing metabolic syndrome (MetS), and this association is greater in women than in men (30). Interestingly, our present study revealed no significant differences in the association between DII and MAFLD in gender.

As a newly proposed diagnosis for metabolic associated fatty liver disorders, MAFLD highlights the role that metabolic anomalies play in the development of the fatty liver. A meta-analysis found that the prevalence of MAFLD was 39.22% globally, with Europe and Asia having the greatest rates of the disease, followed by North America (31). In the entire U.S. population, the prevalence of MAFLD reached 39.1%, based on the findings of a study on the 2017–2018 NHANES in the United States (32). The development and progression of nonalcoholic steatohepatitis (NASH), which can advance to cirrhosis and hepatocellular cancer, may be influenced by the underlying condition of metabolic stiffness associated with MAFLD (33). This poses a substantial risk to human health and places a significant economic burden on society. Multiple genetic, metabolic, and inflammatory factors, including polymorphisms in PNPLA3, Il148M, and TM6SF2, impact the pathogenic pathways mediating the development and progression of MAFLD (34). Moreover, MAFLD is a hepatic phenotype of systemic insulin resistance (IR) (35, 36). Disorders of systemic energy metabolism, including IR and atherogenic dyslipidemia, are closely associated with an increased risk of MAFLD (37). Lipotoxicity, oxidative stress, mitochondrial dysfunction, and endoplasmic reticulum stress would all lead to inflammatory and immune dysfunction in the liver, which could result in harm to hepatic cells and death (38). In NAFLD that progresses, inflammation plays a crucial role, and regulating the immune system is a possible treatment approach. Simultaneously, clinical trials have had disappointing outcomes, and therapeutic care to date has been limited to lifestyle measures (39).

It is commonly recognized that diet quality or patterns are crucial in controlling chronic inflammation (40, 41). Through inflammatory pathways, it has been demonstrated that an excessive consumption of pro-inflammatory and a deficiency in anti-inflammatory food components might hasten the development of NAFLD (42). The DII, which illustrates the inflammatory effects of daily diets, relates to the possibility of a variety of harmful health outcomes (19, 43–45). Zhang et al. (46) reported that DII is related to the development of MetS and has a significant impact on blood pressure, lipid levels, and BMI. Wu et al. (47) investigated the connection between DII and coronary heart disease, indicating that patients with coronary heart disease had considerably higher DII values compared to those without coronary heart disease. Moreover, another study has confirmed that there were higher DII scores in diabetic patients than that in normoglycemic or pre-diabetic groups, indicating that their diets may have a higher ability to cause inflammation [34]; besides, higher DII values in diabetes individuals were associated with a higher likelihood of long-term all-cause and cardiovascular death (48). Deng et al. (49) discovered that among prediabetic participants, an inflammatory diet is related to an increased likelihood of all-cause, cardiovascular disease, all-cancer, and digestive tract cancer death, as evidenced by higher DII scores. In line with previous studies, our research showed that an increase in DII was independently linked to a higher probability of MAFLD, with a 5% rise in the likelihood of MAFLD for each unit of DII value. According to our finding, public health policy should prioritize increasing the availability and affordability of nutrient-dense foods, putting in place educational initiatives to raise public awareness of the relationship between diet and MAFLD risk, and designing settings like offices and schools that encourage the consumption of healthful foods. Furthermore, using this information, medical professionals can better advise patients who may be at risk of developing MAFLD regarding their diet.

The non-linear association between DII and disease has only been studied in a few researches, and one of them found that the risk of heart disease increases quickly when DII is higher than 2 (46). The results of this research, however, revealed that even while MAFLD and DII had a nonlinear relationship, the probability of MAFLD decreased with rising DII score when DII was higher than 3.06. This demonstrated that only when the DII was below 3.06, it could be an independent crisis factor for MAFLD. Additionally, when DII was converted into a categorical variable (quartiles), the findings of the logistic regression used in this study likewise matched the inverted U-shape connection between DII and MAFLD. The association between DII and MAFLD was not statistically significant in comparisons of DII quartiles in the fully adjusted model, despite the *p* value for trend <0.05, indicating a non-linear relationship. To better understand the non-linear relationship and to identify potential confounding factors, further research incorporating larger and more diverse study populations, comprehensive dietary assessments, longitudinal designs, and rigorous adjustment for confounders is warranted.

Our present study has several benefits as follows. Firstly, the sample size and its composition are adequate and representative. Here, 3,633 out of 9,254 US adult populations with MAFLD were involved. Secondly, sample weight was used in this study to make the NHANES participants as close as possible to the actual US population. Thirdly, we used threshold effect analysis to find the nonlinear relationship between DII and MAFLD, so that we could better explain the essence of this two-way relationship. New proof for health policy decisions may be provided by the curve’s shape and inflection point. However, due to a variety of restrictions, there are potential limitations in this study, thus the findings of this study should be considered cautiously. First, as the study was cross-sectional, we were not able to establish a direct correlation between DII and MAFLD development. To track changes in DII scores and their association to the onset and progression of MAFLD, longitudinal studies with high sample numbers are required to follow individuals over time. Second, ultrasound and VCTE was used to diagnose MAFLD, which might have overstated the disease’s prevalence. Although several studies indicate the transient elastography’s incredibly high accuracy (50–52), it cannot be replaced by liver biopsy which is still regarded as the gold standard for diagnosing MAFLD and the severity of liver fibrosis (53, 54). Given the limits of FibroScan, a comprehensive approach that considers multiple diagnostic modalities may be necessary for more accurate and generalizable results. Third, since participants’ medication use was not included in the variables of this research due to the constraints of the NHANES database and considering patients with NAFLD frequently take hepatic steatosis-improving drugs, our results might not accurately reflect the reality. Future studies will need to clarify the potential impacts of medications and address the gaps in NHANES data regarding medication use in MAFLD patients.

## 5 Conclusion

Our study demonstrates that the elevated DII scores were independently associated with incident MAFLD, especially for individuals between the ages of 20 and 41. However, this conclusion applies only to those individuals with DII less than 3.06. The link between DII scores and MAFLD showed a negative correlation for those with a DII higher than 3.06; the precise causes of this need to be further investigated. Our results emphasize the significance of dietary quality in identifying MAFLD-at-risk populations. It will still take more prospective research to confirm our present results.

## Data availability statement

The datasets presented in this study can be found in online repositories. The names of the repository/repositories and accession number(s) can be found at: The NHANES website (accessed on 20 July 2023) provides access to the datasets that were used and/or analyzed during the current investigation.

## Ethics statement

The studies involving humans were approved by the National Center for Health Statistics Research Ethics Review Board granted permission for this survey (protocol number: 2018–01). The studies were conducted in accordance with the local legislation and institutional requirements. The participants provided their written informed consent to participate in this study.

## Author contributions

JY: Data curation, Software, Writing – original draft, Writing – review & editing. JZ: Writing – review & editing. YD: Writing – review & editing. CT: Methodology, Writing – review & editing.
